# The Enhancement of Plant Disease Resistance Using CRISPR/Cas9 Technology

**DOI:** 10.3389/fpls.2018.01245

**Published:** 2018-08-24

**Authors:** Virginia M. G. Borrelli, Vittoria Brambilla, Peter Rogowsky, Adriano Marocco, Alessandra Lanubile

**Affiliations:** ^1^Department of Sustainable Crop Production, Università Cattolica del Sacro Cuore, Piacenza, Italy; ^2^Department of Agricultural and Environmental Sciences – Production, Territory, Agroenergy, University of Milan, Milan, Italy; ^3^Laboratoire Reproduction et Développement des Plantes, Université de Lyon, École Normale Supérieure de Lyon, Université Claude Bernard Lyon 1, Centre National de la Recherche Scientifique, Institut National de la Recherche Agronomique, Lyon, France

**Keywords:** CRISPR/Cas9, crop improvement, genome editing, disease resistance, virus, fungus, bacteria

## Abstract

Genome editing technologies have progressed rapidly and become one of the most important genetic tools in the implementation of pathogen resistance in plants. Recent years have witnessed the emergence of site directed modification methods using meganucleases, zinc finger nucleases (ZFNs), transcription activator-like effector nucleases (TALENs), and clustered regularly interspaced short palindrome repeats (CRISPR)/CRISPR-associated protein 9 (Cas9). Recently, CRISPR/Cas9 has largely overtaken the other genome editing technologies due to the fact that it is easier to design and implement, has a higher success rate, and is more versatile and less expensive. This review focuses on the recent advances in plant protection using CRISPR/Cas9 technology in model plants and crops in response to viral, fungal and bacterial diseases. As regards the achievement of viral disease resistance, the main strategies employed in model species such as *Arabidopsis* and *Nicotiana benthamiana*, which include the integration of CRISPR-encoding sequences that target and interfere with the viral genome and the induction of a CRISPR-mediated targeted mutation in the host plant genome, will be discussed. Furthermore, as regards fungal and bacterial disease resistance, the strategies based on CRISPR/Cas9 targeted modification of susceptibility genes in crop species such as rice, tomato, wheat, and citrus will be reviewed. After spending years deciphering and reading genomes, researchers are now editing and rewriting them to develop crop plants resistant to specific pests and pathogens.

## Introduction

Plant breeding has been the most successful approach for developing new crop varieties since domestication occurred, making possible major advances in feeding the world and societal development. Crops are susceptible to a large set of pathogens including fungi, bacteria, and viruses, which cause important economic losses (FAO, 2017); the enhancement of plant resistance plays an important role in adjusting crop production to meet global population increases. Approaches to disease control that depend on resistant varieties and agrochemicals are usually highly effective whenever they are deployed. However, due to the high evolutionary potential of many plant pathogens, novel genotypes no longer sensitive to the resistance gene or the phytosanitary product can rapidly emerge via mutation or recombination. When this happens, particular disease control approaches can rapidly be rendered ineffective as the novel genotypes increase in frequency through natural selection and quickly spread to other locations, causing failure of control over large geographic areas.

An understanding of interactions between plants and communities of bacteria, fungi, and other microorganisms has been a major area of investigation for many years. The advent of high-throughput molecular technologies has made a more complete inventory of the pathogens associated with particular crops possible, and provided insight into how these communities may be affected by environmental factors and the crop genotype. Disease involves a complex inter-play between a host plant and a pathogen, and the resistance/susceptibility response can involve several components. Natural and induced mutations may change the interaction and inhibit certain steps in the mechanism of infection (Boyd and O’Toole, 2012; Dracatos et al., 2018).

During pre-genomic years, traditional breeding programs were based on the identification of natural and induced mutant alleles for resistance, and their incorporation into elite genotypes through breeding techniques. These approaches were uncertain and imprecise, leading for instance to the transfer of large genome regions instead of just single gene insertions. Nevertheless, mutation breeding methods have been quite successful in improving disease resistance, and traditional plant breeding has been used to generate new crop varieties for decades. Numerous mutants have been developed through mutation induction, showing enhanced resistance to various diseases. Among the most widely known mutants are those induced at the mildew resistance locus (*MLO*) in barley for resistance to powdery mildew (Miklis et al., 2007), and mutations conferring resistance to several lettuce diseases (Christopoulou et al., 2015). The *mlo* mutant is interesting, as the allele has not broken down and has provided unprecedented resistance to mildew for two decades (Panstruga and Schulze-Lefert, 2002). This longevity is due to a gene knockout. In other cases where resistance to specific pathotypes is conferred by a specific host gene allele, mutagenesis needs to be deployed to provide more precise single nucleotide mutations in the target gene sequence.

The revolution driven by the availability of genome and transcriptome sequences offers a new start for plant breeding programs. Association genetics based on single nucleotide polymorphisms (SNPs) and other molecular markers are spreading in plant breeding, creating high throughput data fundamental for the identification of quantitative trait loci (QTL). Major QTL are employed in crops to provide quantitative resistance to pathogens, together with the use of major resistance (*R*) genes introduced into varieties with superior agronomic characteristics.

New breeding techniques (NBTs) are attracting attention in plant research and concern many different areas, such as developmental biology, abiotic stress tolerance or plant-pathogen resistance (Nelson et al., 2018). NBT include the most recent and powerful molecular approaches for precise genetic modifications of single or multiple gene targets. They employ site-directed nucleases to introduce double stranded breaks at predetermined sites in DNA. These breaks are repaired by different host cell repair mechanisms, resulting either in small insertions or deletions via near homologous end-joining (NHEJ) or micro-homology-mediated end-joining (MMEJ), or in a modified gene carrying predetermined nucleotide changes copied from a repair matrix via homologous recombination (HR). Meganucleases (MNs), zinc finger nucleases (ZFNs), transcription activator-like effector nucleases (TALENs), and clustered regularly interspaced short palindrome repeats (CRISPR)/CRISPR-associated protein 9 (Cas9) correspond to the four types of nucleases used in genome editing. The exponential increase in publications reporting the use of CRISPR/Cas9 illustrates the fact that this technology requires less know-how and financial means and has a higher success rate in gene modification compared to the other available nucleases. The application of CRISPR/Cas9 editing has become a powerful tool for future enhancement of agronomic traits in crops (Mohanta et al., 2017).

The objective of this review is to recall the main features of the CRISPR/Cas9 genome editing technique and discuss its application for the enhancement of pathogen resistance in model plants and important crops, with a focus on rice, wheat, and maize.

## CRISPR/Cas9: Advances, Limitations, and New Combinations

**FIGURE 1:**
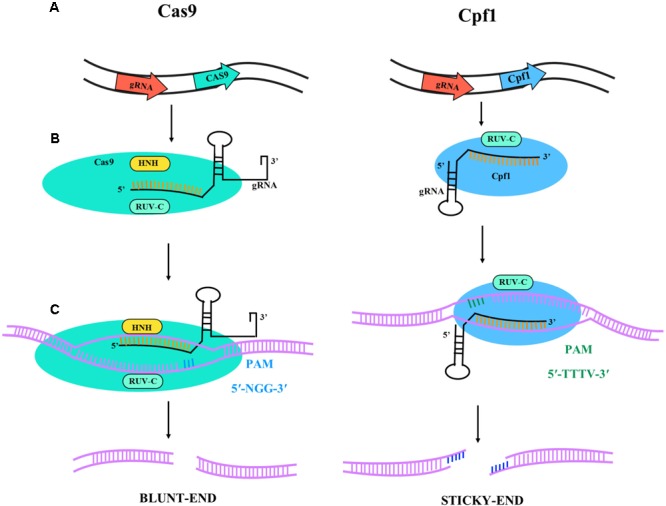
Illustrative diagram of Cas9 and Cpf1 activities. The target specificity is given by the 17-20 nt located at the 5′ end of the sgRNA sequence. **(A)** Primary transcript and gRNA-nuclease (Cas9 or Cpf1) complex formation. The catalytic domains are RUV-C (light blue) and HNH (yellow) for Cas9 and RUV-C for Cpf1. The Cas9 is colored in light blue and the Cpf1 in dark blue; in black is represented the gRNA for gene targeting. **(B)** Gene target activity. Cas9 has 5′-NGG-3′ PAM sequence (blue bars) and Cpf1 has 5′-TTTV-3′ PAM sequence (green bars). **(C)** DNA ends after nuclease activity. Cas9 lead to blunt-end and Cpf1 to sticky-ends.

CRISPR/Cas9 from *Streptococcus pyogenes* (SpCas9) has rapidly assumed an important role in different application areas of plant research and many other fields (Ding et al., 2018; Liu and Moschou, 2018). In the CRISPR/Cas9 system a single-guide RNA (sgRNA) can bind to Cas9 and target it to specific DNA sequences (**Figure 1**). The requirement of a protospacer adjacent motif (PAM) limits the possible target sequences in a gene of interest. This limitation is of minor importance if the aim is simply to inactivate a gene by targeted mutagenesis at any position. It has much more importance for genome editing aiming at the precise change of specific nucleotides in a gene. Consequently, major efforts are under way to find Cas9-like proteins with different PAM sequences or to engineer the original Cas9 from *S. pyogenes* to recognize other PAM sequences. For example, xCas9, an evolved version of SpCas9, has been shown to recognize a broad range of PAM sequences including NG, GAA, and GAT in mammalian cells (Hu et al., 2018). In plants, the most widely explored alternative to SpCas9 is Cpf1 from *Prevotella* and *Francisella* with the PAM sequence TTTV, where “V” is A, C, or G (Endo et al., 2016), and an illustrative diagram is shown in **Figure 1**. Cpf1 is also considerably smaller than Cas9, is capable of RNAse activity to process its guide RNA, and introduces a staggered double break, which can be useful for enhancing homology-directed recombination and generating efficient gene insertion.

## Multiplex Genome Editing: When Does It Become Useful?

The ease of multiplexing, i.e., the simultaneous targeting of several genes with a single molecular construct, is one of the major advantages of CRISPR/Cas9 technology with respect to MN, ZFN, or TALEN. For example, the simultaneous mutation of 14 different genes by a single construct has been demonstrated in *Arabidopsis* (Peterson et al., 2016). In crops, several multiplex genome editing (MGE) strategies were reported early on (Ma et al., 2014; Xing et al., 2014; Zhou et al., 2014; Xu et al., 2016), which were all based on a common strategy, i.e., the assembly of multiple gRNAs under the control of a U3 or U6 promoter into a single construct. In maize, the ISU Maize CRISPR platform (Char et al., 2017) permits the cloning of up to four gRNAs for multiplex gene targeting.

**FIGURE 2:**
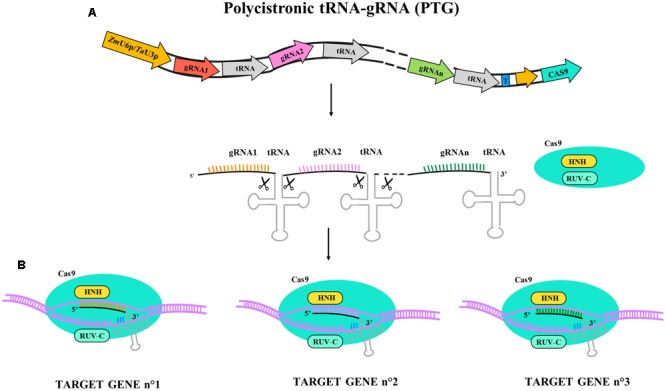
Illustrative diagram of polycistronic tRNA-gRNA (PTG) gene construct and targeting activity for Cas9. PTG is composed of t-RNA-gRNA repeats and is upregulated by ZmU6 promoter or TaU3 promoter according the experimental design as different terminator region (T) are adopted. **(A)** PTG primary transcript. Endogenous endonuclease cuts the tRNA ends and let each tRNA-gRNA targeting the corresponding gene sequence. **(B)** In PTG system more sequence targets are available (n° gene targets) and the different gRNA are represented in different colors (orange, pink, and green).

More recent multiplex systems exploit self-cleavage capacity of RNA molecules containing tRNA sequences. Constructs alternating sgRNA and tRNA sequences under the control of a single U3 or U6 promoter permit reduction of the size of the construct and limit the risk of silencing due to direct repetitions of promoter sequences. The use of such a strategy employing polycistronic tRNA-gRNA (PTG) to generate hereditable mutation in *TaLpx-1* and *TaMLO* genes has been reported in hexaploid wheat (Wang et al., 2018); the PTG system is described in **Figure 2**. Starting from a previous study on gene silencing of *TaLpx-1*, which encodes the wheat 9-lipoxygenase resistance gene to *Fusarium graminearum* (Nalam et al., 2015), the editing of homologs in wheat was tested. The PTG system containing gRNA activity was validated in wheat confirming gene editing efficacy and providing an effective tool for rapid trait pyramiding in breeding programs.

Recently, an alternative approach for MGEs based on PTG has been reported in rice, where crRNA transcription was obtained from introns inserted into Cpf1 and Cas9 sequences (Ding et al., 2018). Multiplex PTG/Cas9 systems can help with multigene family analysis, as reported for the closely related mitogen-activated protein kinase *MPK1* and *MPK6* in rice (Minkenberg et al., 2017). 67% of all lines were double mutants for *MPK* genes with a high frequency of biallelic mutations on multiple target sites. The possibility of programming the PTG/Cas9 to delete chromosomal fragments could be adopted to remove genes and regulatory elements in order to produce transgene free plants.

## Off-Target Mutations: Frequency and Limitations

High specificity is frequently put forward as a major argument in favor of CRISPR/Cas9 technology, for example in comparison to chemically or irradiation-induced mutagenesis. This raises the question of to what extent a gRNA targets only fully complementary genomic DNA sequences, and to what extent other genomic regions (off-target regions) can also be recognized and cleaved by the CRISPR/Cas9 tool, provoking potentially unwanted damage. Two types of off-target effects are evoked by scientists and regulatory agencies: (i) expected off-target in genome regions with high sequence similarity to the target and (ii) unexpected off-target in unrelated genome regions. The former is generally addressed by PCR amplification and sequencing of regions known to be similar to the target, the latter by whole genome sequencing (Feng et al., 2016).

Genome sequence information is necessary for the prediction of expected off-target effects. The search focuses on the 20 bp target sequence involved in base pairing with the gRNA but excludes the PAM 5′-NGG-3′. The PAM functions as a recognition site outside of the targeted element and does not give specificity for nuclease cleavage (Shah et al., 2013). Moreover, the CRISPR/Cas9 system accepts at least three mismatches in the 20 bp DNA target sequence. Most CRISPR/Cas9 design tools take this into account and propose only specific gRNA designs that do not bind theoretical off-target sites with more than 17 bp identity anywhere else in the genome. Such state-of-the art design is effortless if the gene is unique in the genome, but it becomes rather challenging if the gene has one or more paralogs. This also means that the design is generally easier for diploid genomes without recent duplications than for recently duplicated or polyploid genomes. *In silico* genome analysis of potential target sequences in dicots and monocots has confirmed that, as expected, larger genomes contain more PAMs and more potential targets (Bortesi et al., 2016). High specificity of between 87.3% and 94.3% was observed in relatively simple genomes of *Arabidopsis*, rice, tomato, and soybean, whereas maize, a recent allotetraploid with high levels of repetitive DNA, revealed only 29.5% specific targeting (Bortesi et al., 2016).

Analysis of expected off-target sites, with only one to several mismatches with the primary target, has revealed that the position of the mismatches in the sequence is significant. Mismatches in the seed sequence (“seed” is defined as the 12 bp close to the PAM) are generally not supported or poorly supported by the sgRNA/Cas9 complex (Tsai et al., 2015), causing mutation less frequently at off-target sites, although in some cases mutations have been observed, as in barley (Lawrenson et al., 2015), soybean (Jacobs et al., 2015), and rice (Xie and Yang, 2013). Unwanted off-target mutations become more frequent when mismatches are located far from the seed region (Zhang et al., 2014).

To clarify the off-targeting issue in crops, recent investigations have screened progenies of CRISPR/Cas9 knockout in polyploid species. A study of CRISPR mutation frequency and mutation heritability of *TaGW2, TaLpx-1*, and *TaMLO* genes in the allohexaploid wheat was conducted (Wang et al., 2018). The results were different for the three genes: highly conserved for *TaGW2* (target sequence was specific for all three genomes), moderate for *TaLpx-1* (target sequence specific in two genomes), and low for *TaMLO*. The study showed the flexibility of CRISPR/Cas9 technology for implementing complex gene editing where the majority of genes have more than three homologous copies. Also, the gene editing process was investigated across generations: new mutant variants were recovered across multiple gene targets suggesting the transgenerational activity of CRISPR/Cas9 (Wang et al., 2018).

Another study on target accuracy and efficiency was performed in rice on paralogs *OsBEIIb* and *OSBEIIa* (Baysal et al., 2016). The study reveals the discrepancy in gRNA prediction and mutagenesis efficiency, confirming that gRNA with low predicted efficiency can achieve high mutation frequency even though the prediction suggested different targets with high mutagenesis scores. Empirical testing seems necessary in order to avoid putative gRNA inefficiency. Moreover, the authors also investigated off-target mutagenesis, reporting no mutation in the *OSBEIIa* paralog when only *OsBEIIb* was targeted, confirming the high accuracy of the strategy. CRISPR accuracy has been shown also in tomato (Čermák et al., 2015; Pan et al., 2016; Nekrasov et al., 2017).

To conclude, the CRISPR/Cas9 complex can bind with lower efficiency sequences with one to three mismatches. Therefore, expected off-target mutations do occur but can be avoided by rigorous design of the CRISPR/Cas9 tool. Unexpected off-target mutations do not occur at a frequency above the spontaneous mutation rate of plants.

## Plant Transformations: Conventional and Alternative Techniques

The bottleneck in the application of CRISPR/Cas9 technology to a wide range of crops is clearly the regeneration of fertile plants from the cells into which the CRISPR/Cas9 tool has been introduced (Altpeter et al., 2016). Consequently, the efficiency of the entire process remains very species- and genotype-dependent, meaning that in many crop species only a few lab varieties are accessible to CRISPR/Cas9 technology. Other important parameters are the quality of the design of the CRISPR/Cas9 tool and the method chosen for its introduction into the plant cell. As in conventional transgenesis, the introduction of the CRISPR/Cas9 tool can be achieved by the *Agrobacterium-*mediated and biolistic transformation of explants, or by direct transformation of protoplasts. The latter two systems have the advantage that not only can the DNA coding for Cas9 and the sgRNA be transferred, but this also applies for ribonucleoproteins (RNPs), i.e., an *in vitro* assembled complex of Cas9 protein with an sgRNA (Malnoy et al., 2016; Svitashev et al., 2016; Liang et al., 2017), or intermediate versions such as a DNA or RNA coding for Cas9 and an RNA representing the sgRNA (Svitashev et al., 2015; Zhang et al., 2016). In addition, both biolistics and direct DNA transfer permit an increase in the ratio of repair matrix DNA over DNA encoding Cas9 and sgRNA readily, thereby favoring HR over NHEJ/MMEJ.

In maize, ISU Maize CRISPR is a high efficiency public platform using *Agrobacterium-*mediated transformation (Char et al., 2017). The main genotypes used for immature embryo transformation are A188, A634, H99, W117 (Ishida et al., 2007), B104 and the hybrid Hi-II (Char et al., 2017). Private companies seem to prefer biolistic transformation to *Agrobacterium*-mediated transformation in the case of gene editing with donor template (Shi et al., 2017), particularly where multiple copies of donor template DNA molecules can be delivered (Svitashev et al., 2015). Even though both transformation processes have decent efficiencies nowadays, they remain limited to the above genotypes with poor agronomic traits. This limitation has recently been overcome by the overexpression of *Baby boom* (*Bbm*) and *Wuschel2* (*Wus2*) genes, which stimulated callus growth and increased the overall transformation frequency in maize, including in recalcitrant genotypes. Proof of concept has also been provided for enhanced transformation in sorghum (Lowe et al., 2016).

In rice, most genotypes can easily be transformed both via *Agrobacterium*-mediated transformation and by biolistic methods. In order to achieve CRISPR-mediated HR the DNA template is normally introduced via the biolistic method to increase its copy number in the host (Baysal et al., 2016). As for maize, but involving a higher number of studies, protoplast transient assay is becoming an efficient tool for testing CRISPR-target before starting the transformation of embryos or scutellum derived calli by *Agrobacterium* or particle bombardment (Gao et al., 2013; Jiang et al., 2013; Xie and Yang, 2013; Zhou et al., 2014; Lowder et al., 2015; Li et al., 2016; Luo et al., 2016; Wang et al., 2016). Regeneration of rice protoplasts is still very challenging, but important optimization efforts may render it feasible in the near future. In wheat, although very high *Agrobacterium*-mediated transformation efficiencies of up to 90% have been reported for specific wheat genetic backgrounds (Ishida et al., 2015a,b), particle bombardment has been more widely accepted as the standard method in wheat genetic transformation (Hakam et al., 2015; Wang et al., 2018). Remarkable success has been achieved by particle bombardment of both immature embryos and callus cells to obtain transient expression of the CRISPR/Cas9 DNA, and transgene-free homozygous mutant T0 plants have been generated in the absence of any selection (Zhang et al., 2016). Three studies have reported CRISPR mutagenesis in barley by using *Agrobacterium*-mediated transformation of immature embryos (Lawrenson et al., 2015; Holme et al., 2017; Kapusi et al., 2017), while in Kapusi et al. (2017) a comparison with particle bombardment was carried out. Higher numbers of mutants were reported with the *Agrobacterium*-mediated compared to the biolistic transformation approach.

In conclusion, although preferences for certain delivery methods exist for certain species, efficiency is not only linked to the technique itself, but also to the know-how of a given lab as regards a given technique. Polyethylene glycol (PEG) or electroporation-mediated DNA transient expression in protoplasts have proven very useful for the evaluation of the efficiency of CRISPR/Cas9 designs (Malnoy et al., 2016). The importance of preliminary screens will certainly increase with the foreseeable shift from targeted mutagenesis to repair matrix based genome editing, which will increase the number of events to analyze due to lower efficiency. RNP technology has been established in plants and may help toward exemption from regulatory oversight, but its efficiency needs to be improved to make it a routine tool.

## CRISPR/Cas-Based Strategies Conferring Biotic Resistance

Biotic stresses including viral, fungal, and bacterial diseases are responsible for losses ranging from 20% to 40% of global agricultural productivity (Savary et al., 2012). Conferring host plant resistance to pathogens can reduce the impact of disease on crop development and yield, thereby addressing the challenge of feeding the world’s growing population.

Advances in genome editing tools have opened new ways to achieve the improvement of resistance in crops. In recent years, the CRISPR/Cas system has been employed to respond to several agricultural challenges, including the achievement of improved biotic stress resistance (Arora and Narula, 2017). The application of CRISPR/Cas tools has mainly been explored against virus infection, followed by efforts to improve fungal and bacterial disease resistance. Recent studies demonstrating the power of the CRISPR/Cas technology in establishing resistance to these pathogen categories will be further discussed below.

### Virus Resistance via CRISPR/Cas

Plant viruses are a serious threat to many economically important staple and specialty crops. Based on their genome nature, they are classified into six major groups: double-stranded DNA (dsDNA) viruses with no plant viruses in this group, single-stranded DNA (ssDNA), reverse-transcribing viruses, double-stranded RNA (dsRNA), negative sense single-stranded RNA (ssRNA-), and positive sense single-stranded RNA (ssRNA+) viruses (Roossinck et al., 2015). Most studies involving CRISPR-edited plants for virus resistance have targeted ssDNA geminivirus genomes (Ali et al., 2015, 2016; Baltes et al., 2015; Ji et al., 2015) (**Table 1**).

**Table 1:** CRISPR/Cas9 applications for virus resistance.

Plant species	Virus	Target gene	Gene function	Strategy	Reference
*Nicotiana benthamiana* and *Arabidopsis thaliana*	BeYDV	CP, Rep, and IR	RCA mechanism	*Agrobacterium*-mediated transformation of leaves with Cas9/gRNA expression plasmid vectors	Ji et al., 2015
*Nicotiana benthamiana*	BSCTV	LIR and Rep/RepA	RCA mechanism	*Agrobacterium*-mediated transformation of leaves with Cas9/gRNA expression plasmid vectors	Baltes et al., 2015
*Nicotiana benthamiana*	TYLCV BCTV MeMV	CP, Rep and IR	RCA mechanism	*Agrobacterium*-mediated transformation of leaves with a TRV vector in Cas9 overexpressing plants	Ali et al., 2015
*Nicotiana benthamiana*	CLCuKoV MeMV TYLCV	CP, Rep, and IR	RCA mechanism	*Agrobacterium*-mediated transformation of leaves with a TRV vector in Cas9 overexpressing plants	Ali et al., 2016
*Nicotiana benthamiana*	TuMV	GFP1, GFP2, HC-Pro, CP	Replication mechanism	*Agrobacterium*-mediated transformation of leaves with a TRV vector in Cas13a overexpressing plants	Aman et al., 2018
*Nicotiana benthamiana* and *Arabidopsis thaliana*	CMV TMV	ORF1, 2, 3, CP and 3′UTR	Replication mechanism	*Agrobacterium*-mediated transformation of leaves with FnCas9/gRNA expression binary vectors Floral dipping for *Arabidopsis*	Zhang et al., 2018
*Cucumis sativus*	CVYV ZYMV PRSV-W	eIF4E	Host factor for RNA viruses translation	*Agrobacterium*-mediated transformation of cut cotyledons (without embryo) with Cas9/gRNA binary vectors	Chandrasekaran et al., 2016
*Arabidopsis thaliana*	TuMV	eIF(iso)4E	Host factor for RNA viruses translation	*Agrobacterium*-mediated transformation with Cas9/gRNA recombinant plasmid binary vectors (floral dipping)	Pyott et al., 2016
*Oryza sativa* L. *japonica*	RTSV	eIF4G	Host factor for RNA viruses translation	*Agrobacterium*-mediated transformation of immature embryos with Cas9/gRNA expression plasmid vectors	Macovei et al., 2018

*BeYDV, bean yellow dwarf virus; BSCTV, beet severe curly top virus; TYLCV, tomato yellow leaf curl virus; BCTV, beet curly top virus; MeMV, Merremia mosaic virus; TRV, tobacco rattle virus; CLCuKoV, cotton leaf curl Kokhran virus; TuMV, turnip mosaic virus; CMV, cucumber mosaic virus; TMV, tobacco mosaic virus; CVYV, cucumber vein yellowing virus; ZYMV, zucchini yellow mosaic virus; PRSV-W, papaya ring spot mosaic virus-W; PVX, potato virus X; TCV, turnip crinkle virus; CMV, cucumber mosaic virus; RTSV, rice tungro spherical virus; CP, coat protein; Rep, replication association protein; IR, intergenic region; RCA, rolling-circle amplification; LIR, long intergenic region; GFP1, green fluorescent protein 1; GFP2, green fluorescent protein 2; HC-Pro, helper component proteinase silencing suppressor; ORF, open reading frame; UTR, untranslated terminal repeat; eIF4E, eukaryotic translation initiation factor 4E; eIF4G, eukaryotic translation initiation factor 4G.*

Geminiviridae is a large family of plant viruses causing worldwide crop losses among several important families, such as Cucurbitaceae, Euphorbiaceae, Solanaceae, Malvaceae, and Fabaceae (Zaidi et al., 2016). The virus genome is replicated through a rolling-circle amplification mechanism via a dsDNA replicative form, or by recombination-mediated replication (Hanley-Bowdoin et al., 2013). The most important genus of geminiviruses in economic terms is Begomovirus. Begomoviruses infect dicotyledonous plants via the sweet potato/tobacco/silverleaf whitefly (*Bemisia tabaci*) and are mainly found associated to the phloem of infected plants (Gilbertson et al., 2015). Their genome is organized in one (A, monopartite) or two (A and B, bipartite) components containing a common region of ∼220 bp (Fondong, 2013).

The first two studies focusing on resistance to geminiviruses, beet severe curly top virus (BSCTV) and bean yellow dwarf virus (BeYDV) in model plants *N. benthamiana* and *Arabidopsis* were reported by Baltes et al. (2015) and Ji et al. (2015) (**Table 1**). Ji et al. (2015) screened 43 candidate sgRNA/Cas9 target sites in coding and non-coding regions of the BSCTV genome. All the sgRNA/Cas9 constructs reduced virus accumulation in inoculated leaves at varying levels, but a greater resistance to virus infection was observed in *Nicotiana* and *Arabidopsis* plants showing the highest levels of expression of Cas9 and sgRNAs. Similar findings were described by Baltes et al. (2015), who employed 11 sgRNAs targeting Rep motifs, Rep-binding sites, hairpin, and the nonanucleotide sequence of BeYDV, and reported up to 87% reduction in the targeted viral load in *N. benthamiana.*

Two recent works have also employed a CRISPR/Cas9 approach for achieving resistance to begomoviruses (Ali et al., 2015, 2016) (**Table 1**). Both studies were based on the strategy of expressing the CRISPR/Cas9 system in the host cell nucleus to target and cleave the virus genome during replication. Ali et al. (2015) developed sgRNA molecules delivered via a tobacco rattle virus (TRV) vector into *N. benthamiana* plants stably overexpressing the Cas9 endonuclease. SgRNAs were specific for different tomato yellow leaf curl virus (TYLCV) coding and non-coding sequences, targeting the viral capsid protein (CP), the RCRII motif of the replication protein (Rep) and the intergenic region (IR). All sgRNAs were able to interfere with TYLCV genome sequences, but targeting the stem-loop invariant sequence contained in the IR caused a more significant reduction of viral replication and accumulation. The same CRISPR/Cas9 system was tested for targeting simultaneously the monopartite beet curly top virus (BCTV) and the bipartite Merremia mosaic virus (MeMV), geminiviruses that share the same stem-loop sequence in the IR. The results showed attenuated symptoms for both viruses, demonstrating that mixed infection immunity can be developed via a single sgRNA specific for conserved sequences of multiple viral strains.

Furthermore, Ali et al. (2016) analyzed not only the targeting efficiencies of the CRISPR/Cas9 tool but also the emergence of mutated viruses capable of replication and systemic movement. The CRISPR/Cas9 tool was designed to interfere with different coding and non-coding sequences of cotton leaf curl Kokhran virus (CLCuKoV), MeMV, and different severe and mild strains of TYLCV. The work revealed that when the sgRNA/Cas9 complex edited sites in the coding regions of all viruses, virus variants were generated capable of replicating and moving to escape the CRISPR/Cas9 machinery. Conversely, no novel variants were detected in *N. benthamiana* plants carrying sgRNAs addressing the IR sequences. Even though the NHEJ machinery repaired the double strand breaks caused by the Cas9 protein, the IR-repaired variants generated virus genomes unable to replicate, thus providing a better overall interference with the viral life cycle.

Protection against RNA viruses has seemed more difficult to achieve, since the classical SpCas9 from *Streptococcus pyogenes* only recognizes dsDNA. However, the search for and characterization of related nucleases has led to the discovery of enzymes that can bind to and cut RNA, such as FnCas9 from *Francisella novicida* or LwaCas13a from *Leptotrichia wadei*. A first report demonstrating resistance to RNA viruses (Zhang et al., 2018) (**Table 1**) expressed FnCas9 and RNA-targeting sgRNAs specific for cucumber mosaic virus (CMV) and tobacco mosaic virus (TMV) in *N. benthamiana* and *Arabidopsis* plants. Transgenic plants showed CMV and TMV accumulation reduced by 40–80% compared with control plants. Furthermore, the resistance obtained by expressing the sgRNA-FnCas9 system was quite stable and still active in the T6 generation. Importantly, Zhang et al. (2018) observed that the endonuclease activity of FnCas9 was not required for interference with the CMV genome, whereas its RNA-binding activity was essential, meaning that this particular application of FnCas9 can be considered as a CRISPR interference (CRISPRi) tool, similar to catalytically inactive SpCas9 proteins programmed to mitigate gene expression (Larson et al., 2013). The use of a catalytically inactive variant of FnCas9 has the advantage of limiting the onset of mutated viral variants capable of escaping CRISPR/Cas9. Moreover, in contrast with the previously described interference with geminivirus replication in the nucleus, no nuclear localization signal is necessary for FnCas9, which interferes with the RNA viruses in the cytoplasm.

Similar work has been carried out with Cas13a. Aman et al. (2018) exploited this RNA-guided ribonuclease to manipulate the turnip mosaic virus (TuMV) RNA genome (**Table 1**). Four different viral genomic regions were targeted: two targets in the green fluorescent protein (GFP) region, one in the helper component proteinase silencing suppressor (HC-Pro), and one in the coat protein (CP). The most efficient virus interference was observed with CRISPR RNA editing HC-Pro and GFP2 genes and resulted in a reduced replication and spread of TuMV in tobacco leaves. Furthermore, due to the innate ability of Cas13 to process pre-CRISPR RNA into functional CRISPR RNA, the multiplex targeting of several viral mRNA could be markedly improved through this alternative system (Aman et al., 2018).

All the systems aiming at protection against viruses described so far require the maintenance of a transgene for Cas9 and sgRNA in the genome of the crop plants, rendering them subject to genetically modified organism (GMO) regulation. A second strategy for the achievement of viral disease resistance consists in modifying plant genes that will generate virus resistance traits, to segregate the CRISPR/Cas9 tool and to release non-transgenic mutants in the field (Chandrasekaran et al., 2016; Pyott et al., 2016; Macovei et al., 2018) (**Table 1**). Plant host factors are required by RNA viruses to maintain their life cycle, including the eukaryotic translation initiation factors eIF4E, eIF(iso)4E and eIF4G (Sanfacon, 2015). Chandrasekaran et al. (2016) developed cucumber plants resistant to potyviruses by mutating independently two different sites of the host susceptibility gene *eIF4E* by CRISPR/Cas9. Non-transgenic Cucumis *eif4e* mutant plants were obtained by segregation of the CRISPR/Cas9 tool by three generations of backcrossing, making these plants safe for human consumption and for release into the environment, according to the authors. When challenged with viruses from the Potyviridae family, cucumber vein yellowing virus (CVYV), zucchini yellow mosaic virus (ZYMV), and papaya ring spot mosaic virus-W (PRSV-W), homozygous *eif4e* mutants showed immunity. Conversely, heterozygous knockout plants and non-mutant plants did not reveal any resistance to these viruses.

A similar editing approach was utilized by Pyott et al. (2016) in order to introduce site-specific mutations at the closely related *eIF(iso)4E* locus in *Arabidopsis* plants. Both 1 bp insertions and 1 bp deletions in *eIF(iso)4E* conferred complete resistance to the single-stranded RNA potyvirus (+ssRNA) TuMV and no off-target modification was detected in this study. Furthermore, homozygous T3 *eIF(iso)4E* mutants did not significantly differ in growth and development compared to wild-type plants.

Recently, Macovei et al. (2018) have developed new sources of resistance to rice tungro spherical virus (RTSV) through mutagenesis of *eIF4G* alleles in rice plants. The RTSV-resistant T_2_ plants obtained did not show any detectable mutation in the off-target sites and were negative when tested for the presence of Cas9. Furthermore, after inoculation with RTSV, agronomic parameters such as plant height and grain yield were enhanced in the edited rice plants compared to their wild-type counterparts under glasshouse conditions.

The advantage of knocking out host susceptibility genes is that this is a relatively simple method that renders following the mutation easy. The loss of a host factor required for the viral life cycle is a form of recessive resistance that should be more durable than that of dominant *R* genes because viruses undergo a lower selective pressure, preventing their evolution to hinder host defense mechanisms. A possible disadvantage of the knockout strategy is that it may negatively influence plant vigor, supporting the selection of virus variants breaking the resistance, as already observed in nature (Abdul-Razzak et al., 2009). Pyott et al. (2016) and Macovei et al. (2018) did not observe any significant difference in growth defects between mutants and normal plants, although further investigations should be carried out in order to test the durability of this edited recessive resistance.

### Resistance to Fungi Through CRISPR/Cas

Fungal pathogens are responsible for numerous diseases such as mildew, smut, rust, rot and many more. These diseases not only cause dramatic yield losses annually throughout the world but also compromise the quality of the harvested products. Moreover, mycotoxigenic fungi represent a serious concern due to the production of secondary metabolites known as mycotoxins, which cause severe health problems in humans and animals after exposure to contaminated food and feed. Several strategies have been evolved to enhance fungal resistance in plant species based on the current knowledge of molecular mechanisms implicated in plant-pathogen interaction. Potential candidate genes and gene products involved in plant resistance against fungi have been described, and nowadays these are prime targets for editing through the CRISPR/Cas9 approach.

As previously partially discussed, *MLO* loci have been targeted by RNA-guided Cas9 endonuclease in three different plant species: bread wheat, tomato, and grapevine (Wang et al., 2014; Malnoy et al., 2016; Nekrasov et al., 2017) (**Table 2**). *MLO* encodes a protein with seven transmembrane domains localized in the plasma membrane and is ubiquitously present in monocots and dicots (Acevedo-Garcia et al., 2014). It had previously been reported that *MLO* were susceptibility (*S*) genes and that homozygous loss-of-function mutants had significantly increased resistance to powdery mildew in barley, *Arabidopsis* and tomato (Piffanelli et al., 2004; Consonni et al., 2006; Bai et al., 2008). Bread wheat plants mutated by CRISPR/Cas9 in one (*TaMLO-A1*) of the three *MLO* homeoalleles showed improved resistance to *Blumeria graminis* f. sp. tritici infection, a finding that once again demonstrated the important role of *TaMLO* genes in powdery mildew disease (Wang et al., 2014). In tomato, *SlMlo1*, previously identified as the most important of 16 *SlMlo* genes, was targeted at two sites and a deletion of 48 bp was obtained. The edited plants were self-pollinated in order to generate CRISPR/Cas cassette-free individuals. This new non-transgenic variety, “Tomelo,” was fully resistant to *Oidium neolycopersici*. Furthermore, off-target analysis did not reveal any effect on the genomic regions outside the *SlMlo1* locus (Nekrasov et al., 2017). In grapevine, the molecular feasibility of *VvMLO7* knockout has been demonstrated through CRISPR/Cas9 RNP in protoplasts, but no plants have been regenerated (Malnoy et al., 2016). Parallel experiments with RNAi plants showed that the loss of *VvMLO7* reduced susceptibility to *Erysiphe necator* in grapevine (Pessina et al., 2016).

**Table 2:** CRISPR/Cas9 applications for fungal resistance.

Plant species	Fungus	Target gene	Gene function	Strategy	Reference
*Triticum aestivum*	Powdery mildew (*Blumeria graminis* f. sp. *tritici*)	MLO-A1	Susceptibility (*S*) gene involved in powdery mildew disease	Particle bombardment of immature wheat embryos with Cas9/gRNA expression plasmid vectors	Wang et al., 2014
*Solanum lycopersicum*	Powdery mildew (*Oidium neolycopersici*)	MLO1	Major responsible for powdery mildew vulnerability	*Agrobacterium*-mediated transformation of cotyledons with Cas9/gRNA expression plasmid vectors	Nekrasov et al., 2017
*Vitis vinifera*	Powdery mildew (*Erysiphe necator*)	MLO-7	Susceptibility (*S*) gene involved in powdery mildew disease	PEG-mediated protoplast transformation with CRISPR ribonucleoproteins	Malnoy et al., 2016
*Vitis vinifera*	Gray mold (*Botrytis cinerea*)	WRKY52	Transcription factor involved in response to biotic stress	*Agrobacterium*-mediated transformation of proembryonal masses with Cas9/gRNA expression binary vectors	Wang et al., 2018
*Theobroma cacao*	Black pod disease (*Phytophthora tropicalis*)	NPR3	Regulator of the immune system	*Agrobacterium*-mediated transient transformation of stage C leaves with Cas9/gRNA expression binary vectors	Fister et al., 2018
*Oryza sativa* L. *japonica*	Rice blast disease (*Magnaporthe oryzae*)	SEC3A	Subunit of the exocyst complex	Protoplast transformation with Cas9/gRNA expression binary vectors	Ma et al., 2018
*Oryza sativa* L. *japonica*	Rice blast disease (*Magnaporthe oryzae*)	ERF922	Transcription factor implicated in multiple stress responses	*Agrobacterium*-mediated transformation of embryogenic calli with Cas9/gRNA expression binary vectors	Wang et al., 2016

*MLO, MILDEW RESISTANT LOCUS; NPR3, non-expressor of pathogenesis-related 3; ERF922, ethylene responsive factor.*

The RNP approach has also been used for editing *DIPM-1, DIPM-2*, and *DIPM-4* genes in apple protoplasts in order to confer resistance to fire blight disease (Malnoy et al., 2016). Again, only the molecular analysis attesting mutations has been carried out, not disease assay on regenerated plants. In perennial crops such as grapevine and apple, which take several years to flower, the transient introduction of genome editing tools in protoplasts is particularly interesting, since the segregation of stably integrated CRISPR/Cas9 cassettes by backcrosses would take a lot longer than in annual crops with generation times of only a few months. Secondly, the delivery of Cas9/sgRNA complex as RNP is a rapid approach, making possible the achievement of transformed protoplasts and the evaluation of sgRNA efficiency within 1 or 2 days. Thirdly, no foreign DNA is integrated into the genome and the Cas9/sgRNA complexes can be degraded rapidly during the cell culture regeneration process. Furthermore, even in transient approaches, the employment of plasmids can sometimes cause their undesired integration into the host genome, and the prolonged presence of CRISPR/Cas9 tools in the genome increases the risk of off-target mutations, while the CRISPR/Cas9 RNP shows improved on-target specificity. The drawback of this approach is the need to optimize plant regeneration protocols in order to apply this technology.

An example of the successful protection of grapevine by the CRISPR/Cas9 system is the *VvWRKY52* transcription factor, which was targeted by four gRNAs (Wang et al., 2018) (**Table 2**). About 21% of the transgenic plants showed biallelic mutations and were more resistant to *Botrytis cinerea* compared to the monoallelic mutants. No marked difference was observed in phenotype between wild-type and biallelic mutant plants, confirming the efficiency of the CRISPR/Cas9 strategy in woody plants with long reproductive cycles.

A further strategy to expedite genome editing application in slow generation tree crops is the employment of transient leaf transformation coupled to disease assays as demonstrated in *Theobroma cacao* (Fister et al., 2018) (**Table 2**). The authors reported for the first time the transient introduction of CRISPR/Cas9 components into cacao leaves targeting the *Non-Expressor of Pathogenesis-Related 3* (*NPR3*) gene, a suppressor of the immune system, and obtained leaves with increased resistance to *Phytophthora tropicalis*. This new system of *in vivo* mutagenesis in adult cacao trees is a fast and useful technique for validating sgRNA design and observing CRISPR mutagenized phenotypes. It encouraged the authors to regenerate genome-edited somatic embryos to validate the observed results at whole-plant level.

Plants resistant to rice blast disease were generated through CRISPR/Cas9 by disrupting *OsERF922* and *OsSEC3A* genes in rice (Wang et al., 2016; Ma et al., 2018) (**Table 2**). *Ossec3a* mutant plants disrupted in a putative subunit of a complex involved in exocytosis, revealed a pleiotropic phenotype including improved resistance against *Magnaporthe oryzae*, higher levels of salicylic acid (SA) content and up-regulation of pathogenesis- and SA-related genes, but also dwarf stature (Ma et al., 2018). In contrast, no alteration of a number of agronomic traits was observed in T1 and T2 transgene free plants mutated in the ethylene responsive factor (ERF)922, a transcription factor implicated in multiple stress responses. The mutant plants had a reduced number of blast lesions at both seedling and tillering stages (Wang et al., 2016). Overall, these results demonstrate the powerful and advantageous application of the CRISPR/Cas9 system for crop improvement as regards fungal disease resistance.

### Resistance to Bacteria Through CRISPR/Cas

Among the bacterial species living on earth, just a few hundred are involved in crop damage, which often reveals multiple symptoms of disease (Schloss and Handelsman, 2004). Phytopathogenic bacteria are difficult to control, mainly because of undetected asymptomatic infections and the lack of suitable agrochemicals. Generally speaking, bacteriological plant control is based on prevention and exclusion of the pathogen by using genetic resistance, agronomic practices, and biocontrol agents (Kerr, 2016).

Phytopathogenic bacteria can be classified as crop specific, such as *Clavibacter michiganensis*, which is the causal agent of tomato bacterial ring rot; polyphagous specific, such as *Ralstonia solanacearum*, which causes disease in multiple monocot and dicot species; and “kingdom crosser,” such as *Dickeya dadantii*, an entomo-phytopathogen, which can affect plants and animals.

Relatively few studies (**Table 3**) have been published on the application of the CRISPR/Cas system to counteract crop bacterial diseases. CRISPR/Cas9 mutagenesis of *OsSWEET13* has been performed in rice to achieve resistance to bacterial blight disease caused by γ-proteobacterium *Xanthomonas oryzae* pv. *oryzae* (Zhou et al., 2015). *OsSWEET13* is a susceptibility (*S*) gene encoding a sucrose transporter involved in plant-pathogen interaction. *X. oryzae* produces an effector protein, PthXo2, which induces *OsSWEET13* expression in the host and the consequent condition of susceptibility. In a previous work concerning *OsSWEET14* promoter mutagenesis adopting a TALEN approach, the disruption of this gene rendered the *X. oryzae* effector unable to bind *OsSWEET14* and ultimately resulted in disease resistance (Li et al., 2012). Similarly, Zhou et al. (2015) obtained a null mutation in *OsSWEET13* in order to better explore PthXo2-dependent disease susceptibility, and resultant mutants were resistant to bacterial blight. Further genome editing strategies for multiplexed recessive resistance using a combination of the major effectors and other resistance (*R*) genes will be the next step toward achieving bacterial blight resistance.

**Table 3:** CRISPR/Cas9 applications for bacterial resistance.

Plant species	Fungus	Target gene	Gene function	Strategy	Reference
*Oryza sativa*	Bacterial blight (*Xanthomonas oryzae* pv. *oryzae*)	SWEET13	Sucrose transporter gene	*Agrobacterium*-mediated transformation of embryogenic callus with Cas9/gRNA expression plasmid vectors and TALEN	Li et al., 2012; Zhou et al., 2015
*Citrus paradisi*	Citrus canker (*Xanthomonas citri* subspecies *citric*)	LOB1	Susceptibility (*S*) gene promoting pathogen growth and pustule formation	*Agrobacterium*-mediated transformation of epicotyl with Cas9/gRNA expression plasmid vectors	Jia et al., 2016
*Citrus sinensis* Osbeck	Citrus canker (*Xanthomonas citri* subspecies *citric*)	LOB1	Susceptibility (*S*) gene promoting pathogen growth and pustule formation	*Agrobacterium*-mediated transformation of epicotyl with Cas9/gRNA expression plasmid vectors	Peng et al., 2017
*Malus domestica*	Fire blight (*Erwinia amylovora*)	DIPM-1 DIPM-2 DIPM-4	Susceptibility factor involved in fire blight disease	PEG-mediated protoplast transformation with CRISPR ribonucleoproteins	Malnoy et al., 2016

*CsLOB1, Lateral Organ Boundaries 1; DIPM, DspE-interacting proteins of Malus.*

Two recent works have reported the employment of CRISPR/Cas9 with the aim of producing citrus plants resistant to citrus bacterial canker (CBC). CBC is caused by *Xanthomonas citri* subsp. *citri* (*Xcc*) and is the most widespread disease among commercial cultivars. In the first work, Jia et al. (2016) generated canker resistant mutants by editing the PthA4 effector binding elements in the promoter of the *Lateral Organ Boundaries 1* (*CsLOB1*) gene in Duncan grapefruit. Mutated lines showed a decrease in typical canker symptoms 4 days post inoculation with *Xcc*, and no further phenotypic alterations were detectable. Furthermore, no potential off-target mutations in other *LOB* family genes were found by PCR-sequencing. The second work, by Peng et al. (2017), confirmed the link between *CsLOB1* promoter activity and CBC disease susceptibility in Wanjincheng orange (*Citrus sinensis* Osbeck). The complete deletion of the EBE_PthA4_ sequence from both *CsLOB1* alleles induced resistance enhancement to CBC. Moreover, no alteration in plant development was observed after *CsLOB1* promoter modification. Additional efforts will be required to generate non-transgenic canker-resistant citrus varieties for facilitating their agronomic application in CBC prevention.

## Future Prospects

In an era marked by political and societal pressure to reduce the use of pesticides, crop protection by genetic improvement provides a promising alternative with no obvious impact on human health or the environment. Genome editing is one of the genetic levers that can be adopted, and disease resistance is frequently cited as the most promising application of CRISPR/Cas9 technology in agriculture. There are three main reasons for this: firstly, scientific knowledge of the molecular mechanisms underlying numerous pathosystems is sufficiently advanced to enable the proposal of genes to be edited in order to achieve resistance. Secondly, disease resistance can frequently be achieved by the modification of a single gene, which is technically less challenging. This is similar to the modification of metabolic pathways, where the editing of a single gene can also have an all-or-nothing effect, but different from abiotic stress tolerance, where generally numerous genes have to be modified in a coordinated fashion to achieve incremental improvements. Thirdly, targeted mutagenesis, the only use of CRISPR/Cas9 technology at present mastered with respect to crops, is readily applicable to disease resistance, since the inactivation of susceptibility genes leads to protection. For other agriculturally interesting traits the achievement of positive effects by the loss-of-function of genes is a more delicate matter. However, acting as the spearhead of genome editing in crops also puts a certain responsibility on plant pathologists.

The first challenge is to demonstrate that the promises made by proofs of concept in confined environments can be maintained under field conditions. It is one thing to show that the population of a pathogen or the size of disease lesions is reduced in a greenhouse and another to protect a crop year after year under varying environmental conditions. Field tests are also necessary for correct evaluation of the agronomic fitness of the edited crops. Most of the genes inactivated by CRISPR/Cas9 technology in order to obtain disease resistance are likely to have roles in the physiology of the plant other than that linked to the life cycle of the pathogen. For example, triple knockouts of wheat *TaMLO* were not only resistant to powdery mildew but also showed leaf chlorosis (Wang et al., 2014), whereas EMS-induced triple mutants with non-conservative point mutations in *TaMLO* did not show obvious pleiotropic phenotypes (Acevedo-Garcia et al., 2017). Therefore, encouraging greenhouse observations of plant development or measurements of key parameters such as height, leaf area or grain weight absolutely must be confirmed under field conditions by multi-environmental yield trials in order to measure the relative importance of negative side effects. A final limitation of many published proofs of concept is that they involve lab varieties, which can easily be regenerated after the introduction of Cas9 and sgRNA, but which often have only a limited agronomic value. It remains to be shown that the phenotypic effects are maintained in elite lines under field conditions.

The second challenge is the durability of the disease resistances, and their agronomic management. This challenge needs to be dealt with seriously, in order to convince a public often hostile to this technology. Durability is not a specific aspect of resistance genes obtained by genome editing, and the answers are the same as for introgressed resistance genes discovered in the genetic variability of the species: (i) the stacking of several resistance genes, preferably with different modes of action, (ii) a focus on systems other than NBS-LRR receptor kinases known to break down rapidly, and (iii) good agronomic practices, including, in particular, crop rotation and the concomitant use of biocontrol agents. An example of two independent CRISPR/Cas9-derived resistances against the same disease are the knockouts of *TaMLO* (Wang et al., 2014) and *TaEDR1* (Zhang et al., 2017), both conferring resistance to powdery mildew in wheat. Beyond the creation of novel alleles conferring protection, CRISPR/Cas9 technology can also be helpful in the stacking process itself. In contrast with the introgression of conventional resistance genes, the technology not only avoids genetic drag on neighboring regions with potentially negative impacts on agronomic performance, but also permits the simultaneous creation of multiple resistances in a single generation by multiplexing, i.e., the parallel use of several sgRNAs targeting different genes. Admittedly, multiplexing becomes more challenging with increasing ploidy levels, and in the above example in hexaploid wheat (A, B, and D genome), three *TaMLO* genes and three *TaEDR1* genes would need to be modified in parallel.

The third challenge is to overcome the present technical limitation regarding targeted mutagenesis and to implement true genome editing in crop plants. Targeted mutagenesis introduces random mutations (generally short insertions or deletions) at a predetermined site of a given gene, leading generally to loss-of-function, whereas true genome editing introduces predetermined base changes at one or several specific positions in a gene. For example, the elongation initiation factor 4E (eIF4E) is necessary for the translation of RNA into protein for both the host cell and single-stranded RNA viruses of the Potyviridae family. As described above, loss-of-function of eIF4E by targeted mutagenesis has been achieved in several model and crop species, consistently conferring resistance to potyviruses but also impacting the host plants to varying degrees. The specific modification of amino acids known to be important for the translation of viral but not host proteins would permit driving resistance to potyviruses without affecting plant physiology (Bastet et al., 2017). The expression of a transgene carrying a synthetic allele with six mutations in an *Arabidopsis eif4e* mutant validated the concept (Bastet et al., 2018), demonstrating indirectly the potential benefit of genome editing over targeted mutagenesis. However, at present true genome editing by HR is still hampered by very low efficiencies in plants, although it has recently become routine in many animal species. Continued efforts to improve its efficiency, for example by the use of *lig4* (Endo et al., 2016) or *polQ* mutations (Saito et al., 2017), or a copy number increase of the repair matrix by virus vectors (Čermák et al., 2015), are crucial to increasing the range of tools available to plant pathologists. Base editing, to date permitting C to T and A to G transitions in plants, is more limited in scope but has recently emerged as a readily available alternative for certain editing projects (Zhang et al., 2017; Hua et al., 2018).

The long term success of CRISPR/Cas9 technology in plant protection is dependent on new scientific knowledge. CRISPR/Cas9 technology can only be used if one knows which gene(s) to modify and which modification(s) to carry out in these genes in order to render plants resistant to disease. When pathogen resistance is achieved by the knock-out of one or several genes, inactivating mutations can easily be provoked by CRISPR-mediated specific DNA break and activation of the cell’s error prone DNA repair, based on NHEJ. In this case, CRISPR can be used to target and inactivate a single gene or large gene families, both through single gRNA which matches several targets, or by multiplexing the system by introducing several gRNAs simultaneously. On the contrary, when specific allelic variants are involved in resistance, CRISPR-DNA break can be coupled with the less frequent cell repair mechanism based on HDR. The DNA template for HDR should be introduced into the cell together with the effector nuclease. This permits the introduction of a custom-designed sequence into the genome. The use of HDR, compared to NHEJ, can indefinitely expand the possibility of the type of mutations inserted by CRISPR, as any sequence can be inserted into a site of choice. Nevertheless, HDR is still technically challenging due to its low efficiency, the difficulty of having a selective marker and the lack of multiplexing protocols. These are aspects that will need to be improved if CRISPR applications are to expand in plant breeding. Despite the recent judgment of the Court of Justice of the European Union issued that organisms created using genome editing techniques are to be regulated as GMOs (Callaway, 2018), anyhow continuous efforts in plant pathology are necessary, in order to identify and characterize the genes involved in plant pathogen interactions. For example, the past decade was marked by the discovery of hundreds of effector molecules that are synthesized by different classes of pathogens and transferred into the host cell. A major challenge is to identify the host proteins targeted by these effectors and to characterize the underlying genes, which are one of many possible targets for future genome editing approaches. New knowledge does not necessarily have to stem from the crop species of interest. For example, the targeted mutagenesis of wheat *TaMLO* was based on knowledge of another crop, barley, where *Hvmlo* mutant varieties have provided good protection against powdery mildew that has not yet broken down, and the modification of *TaERF1* exploited knowledge from the model species *Arabidopsis*. These examples perfectly illustrate the added value of genome editing, which permits the enlargement of the gene pool of a crop species beyond all the available natural variability, by means of the transfer of knowledge acquired in other crops or model species.

## Author Contributions

VGB contributed by writing and editing the major part of the review. AL, AM, and PR organized and prepared some of the parts of this review. VB and PR critically revised the manuscript. AM and AL contributed to the design of the work’s layout and were responsible for obtaining final approval from the other contributors.

## Conflict of Interest Statement

The authors declare that the research was conducted in the absence of any commercial or financial relationships that could be construed as a potential conflict of interest.
